# Electrolyte disorders and aging: risk factors for delirium in patients undergoing orthopedic surgeries

**DOI:** 10.1186/s12888-016-1130-0

**Published:** 2016-11-23

**Authors:** Li-Hong Wang, Dong-Juan Xu, Xian-Jiao Wei, Hao-Teng Chang, Guo-Hong Xu

**Affiliations:** 1 Department of Orthopedics, Dongyang People’s Hospital, Wenzhou Medical University, 60 Wuning West Road, 322100 Dongyang, People’s Republic of China; 2 Department of Psychiatry, Dongyang People’s Hospital, Wenzhou Medical University, Dongyang, People’s Republic of China; 3 Department of Medical Records, Dongyang People’s Hospital, Wenzhou Medical University, Dongyang, People’s Republic of China; 4 Graduate Institute of Biomedical Science, College of Medicine, China Medical University, Taichung City, 40402 Taiwan; 5 Department of Computer Science and Information Engineering, Asia University, Taichung City, 41354 Taiwan

**Keywords:** Orthopedic surgery, Postoperative delirium, Age-related risk factor, Electrolyte disorders

## Abstract

**Background:**

At present, the exact mechanism of postoperative delirium has not been elucidated. The purpose of this study was to analyze the incidence of delirium in patients undergoing orthopedic surgeries and to explore possible related factors.

**Methods:**

This is a retrospective study. We used 582 patients who had undergone orthopedic surgery between January 2011 and December 2014. The surgeries consisted of 155 cases of internal fixation for intertrochanteric fracture (IFIF), 128 cases of femoral head replacement (FHR), 169 cases of total hip arthroplasty (THA) and 130 cases of total knee arthroplasty (TKA). Among the 582 patients, 75 developed postoperative delirium (an incidence of 12.9%). The demographics of the patients, which included age, gender, operation duration and blood loss, were statistically analyzed with univariate logistic regression analysis and then multivariate logistic regression. To investigate the influences of different electrolytes disorders for postoperative delirium, the Chi-square test was used.

**Results:**

Multivariate logistic regression analysis indicated that postoperative delirium incidence in patients aged 70–79 years and in patients aged ≥80 years was higher than that in patients aged <70 years, odds ratio (OR) values were 6.33 and 26.37, respectively. In addition, the incidence of postoperative delirium in the group of patients with electrolyte disorders was higher than that in the normal group (OR, 2.38). There were statistically significant differences between the delirium group and the non-delirium group in the incidences of the sodium and calcium disorders.

**Conclusions:**

Aging and postoperative electrolyte disorders (hyponatremia and hypocalcemia) are risk factors for postoperative delirium in patients undergoing orthopedic surgeries.

## Background

Delirium is the most common mental disorder encountered in older patients. However, it is difficult to diagnose because of no unified diagnostic methods and crypticity of postoperative delirium. In different hospital units, the incidence of delirium varied from 10% in emergency departments [1] to 70% in intensive care units [2]. The higher figures were associated with frail patients or those with complex surgeries [3]. Postoperative delirium is an acute central nervous system dysfunction after surgical stress that can include clinical features such as acute and non-specific disturbance of consciousness, attention, cognitive ability and sleep-wake cycles [4]. In orthopedic wards, previous studies indicated that delirium most often occurs after surgeries for hip fracture or hip replacement [5, 6]. Recently, however, Fineberg et al. [7] and Chung et al. [8] reported postoperative delirium in patients with spine and knee replacement surgeries. This suggested that postoperative delirium may be a common phenomenon in patients undergoing orthopedic surgeries. Postoperative delirium leads to a delay in recovery, extended hospitalization and increased medical costs. It can also lead to bedsores and fall-related fractures. These complications can substantially affect the patient’s rehabilitation process and quality of life [9, 10].

At present, there is no standard procedure for the prevention and treatment of postoperative delirium. Gleason reported that donepezil was effective for the treatment of postoperative delirium [11], but in contrast, the study of Sampson et al. found that donepezil could not significantly reduce the incidence of postoperative delirium or shorten the resulting period of hospitalization [12]. Low-dose haloperidol and olanzapine are probably effective for the treatment of postoperative delirium but not for its prevention [13]. Theoretically, early prevention based on etiology can reduce the incidence of postoperative delirium or may reduce the harm of its effects. However, the etiology and the mechanisms of postoperative delirium are still unclear [14]. Several previous studies have shown that fluid/electrolyte disorders are closely related to postoperative delirium [7, 15, 16], but the influence of different electrolytes on postoperative delirium remains controversial.

In this study, we retrospectively selected 582 patients who had undergone one of four different orthopedic surgeries, including internal fixation for intertrochanteric fracture (IFIF), femoral head replacement (FHR), total hip arthroplasty (THA) and total knee arthroplasty (TKA). Using logistic regression analyses, we looked for specific risk factors among this population and investigated the mechanism of postoperative delirium. In addition, we tried to find clinical evidence for the prevention and treatment of postoperative delirium.

## Methods

### Ethical considerations

Ethical approval was obtained for this retrospective study from the Internal Review Board of Dongyang People’s Hospital. According to the decision of the Internal Review Board, informed consent was not necessary for this retrospective chart review.

### Study design and participants

As a retrospective study, the information described in this paper was obtained from medical chart review. Between January 2011 and December 2014, 695 individuals who underwent one of four orthopedic surgeries (IFIF, FHR, THA and TKA) were enrolled. Among the 695 cases, 113 were excluded because of 20 cases of emergency operation or simultaneous bilateral surgery; 56 cases with mental illness, psychiatric drugs or preoperative cognitive impairment such as dementia; 14 cases with severe vision or hearing disorders who could not complete the cognitive function tests; 11 cases with cerebral infarction or cerebral hemorrhage and 12 cases who were lost to follow-up or for whom the follow-up data were not complete. Thus, a total of 582 cases, including 155 cases of IFIF, 128 cases of FHR, 169 cases of THA and 130 cases of TKA, were finally analyzed in this study.

The 582 cases were divided into two groups: a non-delirium group and a delirium group. The non-delirium group comprised 507 cases and the delirium group comprised 75 cases. According to the medical chart review, a senior psychiatry physician had independently made a diagnosis decision based on the DSM-IV-TR criteria [17] and proposed a treatment plan for ever delirium cases.

### Outcome evaluation

Demographic data including age, gender and blood type were collected. Perioperative factors including duration of hospitalization, type of surgery, anesthesia, preoperative hospitalization, preoperative comorbidities, preoperative and postoperative albumin, preoperative and postoperative hemoglobin, postoperative electrolyte disorders, blood loss, operation time, postoperative intensive care unit (ICU) care and blood transfusion were evaluated through a retrospective chart review. In this study, the preoperative comorbidities were limited to diabetes, chronic obstructive pulmonary disease, pulmonary infections, hypertension and cardiac dysfunction, and the postoperative electrolyte disorders were limited to the higher or lower level than normal for sodium, potassium, chlorine, calcium (Table 1). Clinical laboratory data including the level of albumin and hemoglobin were measured preoperatively and 1 day postoperatively, sodium, potassium, calcium and chlorine were measured 1 day postoperatively. In addition, perioperative blood loss was measured based on hemoglobin levels and weight.

**Table 1:** Electrolyte disorders

	Disorder(mmol/L)	Normal (mmol/L)
Sodium	<135 OR >145	135 ~ 145
Potassium	<3.5 OR >5.5	3.5 ~ 5.5
Chlorine	<95 OR >105	95 ~ 105
Calcium	<2.25 OR >2.58	2.25 ~ 2.58

### Statistical analysis

Continuous variables are presented as the mean ± standard derivation (SD), and a Student’s *t*-test was used to determine whether there was a significant difference between the two groups (Table 2). Continuous variables with significant differences were transformed into dummy variables. To examine the effect of demographic and perioperative factors on the development of postoperative delirium, univariate logistic regression was employed first to identify the effective, correlated items, followed by multivariate logistic regression analyses with Enter Method in SPSS. To investigate the influences of different electrolytes disorders for postoperative delirium, the Chi-square test was used for the comparison of categorical variables (Table 5). All statistics were performed with SPSS version 18.0, and the level of significance was set at *P* < 0.05.

**Table 2:** Statistical analysis of continuous variables between the delirium group and the non-delirium group

	Delirium	Non-delirium	*t* ^a^	*P*-value
Cases	75	507	–	–
Duration of hospitalization (d)	21.25 ± 8.45	20.85 ± 8.34	0.42	0.68
Preoperative Albumin (g/L)	38.23 ± 3.34	39.99 ± 4.37	−3.38	0.00
Postoperative Albumin (g/L)	30.12 ± 4.46	31.49 ± 3.78	−1.89	0.07
Preoperative Hemoglobin (g/L)	114.98 ± 14.56	125.18 ± 18.32	−3.72	0.00
Postoperative Hemoglobin (g/L)	102.21 ± 15.34	106.98 ± 17.84	−1.60	0.14

^a^A Student’s *t*-test was used to analyze these variables

## Results

### Incidence and onset of delirium

Among the 582 cases of individuals undergoing orthopedic surgery, there were 75 cases of postoperative delirium, an incidence of 12.9%. The onset time of postoperative delirium ranged from 7 h to a maximum of 16 days after the operation, with an average of 2.8 days postoperation. Most delirium patients showed improvement within 1 week of treatment using haloperidol and regaining electrolyte balance. No mortality during hospitalization occurred because of the postoperative delirium. The incidences of postoperative delirium varied among patients with different surgeries: 17.4% (27/155) for IFIF, 25% (32/128) for FHR, 5.9% (10/169) for THA and 4.6% (6/130) for TKA.

### Student’s t-test for the continuous variables

In the comparison with the non-delirium group, patients with postoperative delirium had significantly lower levels of preoperative albumin and hemoglobin (*P* < 0.01, Table 2). Preoperative albumin and hemoglobin were then transformed into dummy variables (Table 3) and were used in the univariate logistic regression analysis.

**Table 3:** Univariate logistic regression analysis for postoperative delirium

Characteristic	*B* ^a^	S.E.^b^	Wald	*P*-value	OR^c^	95% CI^d^
Gender						
Male	–	–	–	–	1.000	–
Female	−0.053	0.256	0.043	0.835	0.948	(0.574, 1.566)
Age (years)			38.093	<0.001		
<70	–	–	–	–	1.000	–
70–79	2.709	0.738	13.478	<0.001	15.007	(3.534, 63.724)
≥80	3.819	0.733	27.126	<0.001	45.540	(10.822,191.637)
Blood type			4.949	0.176		
A	–	–	–	–	1.000	–
B	−0.234	0.330	0.500	0.479	0.792	(0.414, 1.513)
AB	−1.070	0.511	4.383	0.036	0.343	(0.126, 0.934)
O	−0.020	0.304	0.004	0.947	0.980	(0.540, 1.779)
Type of surgery			29.940	<0.001		
IFIF	–	–	–	–	1.000	–
FHR	0.458	0.294	2.420	0.120	1.580	(0.888, 2.813)
THA	−1.210	0.389	9.689	0.002	0.298	(0.139, 0.639)
TKA	−1.472	0.469	9.872	0.002	0.229	(0.092, 0.575)
Anesthesia						
General anesthesia	–	–	–	–	1.000	–
Epidural anesthesia	0.265	0.560	0.224	0.636	1.304	(0.435, 3.909)
Preoperative hospitalization (d)			32.560	<0.001		
1–3	–	–	–	–	1.000	–
4–6	1.522	0.294	26.807	<0.001	4.580	(2.575, 8.148)
≥7	0.175	0.372	0.223	0.637	1.192	(0.575, 2.469)
Preoperative comorbidities			6.004	0.111		
0	–	–	–	–	1.000	–
1	0.302	0.291	1.075	0.300	1.352	(0.764, 2.392)
2	0.162	0.357	0.205	0.650	1.176	(0.584, 2.369)
≥3	1.107	0.459	5.825	0.016	3.026	(1.231, 7.436)
Preoperative albumin (g/L)			14.368	0.001		
<35	–	–	–	–	1.000	–
35–39	0.000	0.341	0.000	1.000	1.000	(0.513, 1.950)
≥40	−1.061	0.379	7.838	0.005	0.346	(0.165, 0.727)
Preoperative hemoglobin (g/L)						
Anemic (male, <120; female, <1 l0)	–	–	–	–	1.000	–
Normal (male, ≥120; female, ≥110)	−0.607	0.262	5.343	0.021	0.545	(0.326, 0.912)
Postoperative electrolyte disorders						
No	–	–	–	–	1.000	–
Yes	0.663	0.263	6.354	0.012	1.941	(1.159, 3.249)
Blood loss (mL)			3.905	0.142		
<200	–	–	–	–	1.000	–
200–399	−0.260	0.264	0.971	0.324	0.771	(0.460, 1.293)
≥400	−0.866	0.456	3.606	0.058	0.421	(0.172,1.028)
Operation time (min)			19.298	<0.001		
<60	–	–	–	–	1.000	–
60–89	−0.345	0.333	1.074	0.300	0.708	(0.369, 1.360)
≥90	−1.666	0.417	15.963	<0.001	0.189	(0.084, 0.428)
Postoperative ICU care						
No	–	–	–	–	1.000	–
Yes	1.005	0.256	15.443	<0.001	2.731	(1.655, 4.507)
Blood transfusion (mL)			5.618	0.060		
<600	–	–	–	–	1.000	–
600–1199	0.666	0.281	5.618	0.018	1.947	(1.122, 3.379)
≥1200	0.290	0.333	0.758	0.384	1.336	(0.696, 2.564)

^a^Regression coefficient

^b^Standard error

^c^Odds ratio

^d^95% confidence interval

### Univariate logistic regression analysis

To carry out a univariate logistic regression analysis, postoperative delirium was set as the dependent variable, and the independent variables consisted of 14 factors including preoperative albumin, preoperative hemoglobin, age, gender, blood type, type of surgery, anesthesia, preoperative hospitalization, preoperative comorbidities, postoperative electrolyte disorders, blood loss, operation time, postoperative ICU care and blood transfusion (Table 3). Univariate logistic regression analysis showed that eight factors were associated with postoperative delirium including age, type of surgery, preoperative hospitalization, preoperative albumin, preoperative hemoglobin, postoperative electrolyte disorders, operation time and postoperative ICU care (*P* < 0.05, Table 3).

### Multivariate logistic regression analysis

The eight factors that were associated with delirium based on the results shown in Table 3 were then used in a multivariate logistic regression analysis. This analysis indicated that aging and postoperative electrolyte disorders were the risk factors for postoperative delirium in patients undergoing orthopedic surgeries (Table 4). The incidences of postoperative delirium in the group of individuals aged 70–79 and in the group of individuals ≥80 years old were higher than that in the group of individuals aged <70. The incidence of postoperative delirium in individuals with electrolyte disorders was higher than that in individuals with normal electrolyte control (Table 4).

**Table 4:** Multivariate logistic regression analysis for postoperative delirium

	*B* ^a^	S.E.^b^	Wald	*P*-value	OR^c^	95% CI^d^
Age (years)			24.022	<0.001		
<70	–	–	–	–	1.000	–
70–79	1.845	0.788	5.477	0.019	6.328	(1.350, 29.667)
≥80	3.272	0.808	16.414	<0.001	26.371	(5.415, 128.416)
Postoperative electrolyte disorders						
No	–	–	–	–	1.000	–
Yes	0.865	0.367	5.556	0.018	2.376	(1.157, 4.879)

^a^Regression coefficient

^b^Standard error

^c^Odds ratio

^d^95% confidence interval

### The influence of different electrolytes for postoperative delirium

The analysis of electrolytes indicated that there were statistically significant differences between the two groups in the incidences of the sodium and calcium disorders (Table 5). All of the postoperative calcium and sodium disorders were hypocalcemia and hyponatremia, respectively except two cases with hypernatremia.

**Table 5:** The influence of different electrolytes disorders for postoperative delirium

	Delirium	Non-delirium	*P-value*	OR^a^	95% CI^b^
Cases	75	507	–	–	–
Potassium disorder	8 (10.7%)	27 (5.3%)	0.07	2.123	(0.926, 4.865)
Sodium disorder	20 (26.7%)	54 (10.7%)	0.00	3.051	(1.700, 5.472)
Chlorine disorder	3 (4.0%)	53 (10.5%)	0.08	0.375	(0.109, 1.173)
Calcium disorder	42 (56.0%)	198 (39.1%)	0.01	1.986	(1.217, 3.241)

^a^Odds ratio

^b^95% confidence interval

## Discussion

Postoperative delirium is a common phenomenon in older individuals, and it causes recovery delay, extended hospitalization and increases in medical costs. It can have a notable effect on an individual’s quality of life. Many previous studies have tried to explain the etiology and the mechanisms of postoperative delirium [14, 18, 19]. However, there is no standard procedure for the prevention or treatment of postoperative delirium. In this study, we showed that aging and postoperative electrolyte disorders were risk factors for postoperative delirium in patients undergoing orthopedic surgeries.

The reported incidence of postoperative delirium varies widely. We speculate that a possible reason for this variability is that the diagnostic methods for postoperative delirium are not uniform. The incidence of postoperative delirium as previously reported ranges from 3.1 to 62%, with the higher incidence in patients after coronary artery bypass surgery [20] and the lower incidence occurring in patients after TKA [8] and spinal surgery [21]. We hypothesize that these differences may be related to diagnostic methods, diagnostic accuracy, sample size and types of surgery. At present, many different diagnostic methods for postoperative delirium have been used. The Confusion Assessment Method (CAM), the CAM for the ICU and the Diagnostic and Statistical Manual of Mental Disorders, Fourth Edition are the most often used diagnostic methods [18]; however, diagnostic criteria are not uniform. In addition, the variability in the individual who assesses delirium—psychiatrists, nurses, or the researchers—could also lead to the perception of different incidences. We believe that it is very important to have a senior psychiatry physician independently make the diagnostic decision. Furthermore, unlike most past research, the present study broadly investigated the incidence of postoperative delirium after four types of common orthopedic surgery, not just a single type of surgery. All these measurements might be helpful to the accuracy of diagnosis.

Although previous studies reported that many factors might be associated with postoperative delirium, such as Alzheimer disease (AD), preoperative hospitalization, anesthesia, diabetes, hemoglobin level, blood loss, operating time and blood transfusion [5, 8, 21], aging was the factor most often noted in the risk prediction models [18]. Our investigation supports this result. Although the role of postoperative electrolyte disorders in postoperative delirium is still debatable [15, 22, 23], the presence of an electrolyte disorders or an abnormal electrolyte channel is associated with many neuropsychiatric disorders including AD [24], dementia [25] and depression [26]. Cisternas et al. found that an increased potassium intake can improve cognitive performance and might be important in the prevention of AD onset [27]. Cherbuin’s study confirmed that higher intakes of potassium, calcium and magnesium are associated with a reduced risk of developing vascular dementia [28]. Therefore, we have reasons to propose that an electrolyte disorders may be a risk factor for some psychiatric disorders, including postoperative delirium.

Several previous studies have shown that fluid/electrolyte disorders are closely related to postoperative delirium [7, 15, 16], but the influence of different electrolytes on postoperative delirium remains controversial. Some studies have suggested that a disturbance in potassium or sodium levels might be a factor in postoperative delirium [16, 29], but Caplan’s study [19] found that significantly lower serum levels of magnesium and phosphate, not potassium, were associated with delirium. Thus, the exact mechanism by which electrolyte disorders contribute to the occurrence of postoperative delirium is unclear. According to the delirium guideline [30], an electrolyte disorder is a very plausible risk factor for postoperative delirium, and a disturbance in sodium or potassium levels is usually associated with a disorder of body fluids, including hypotonic or hyperosmotic dehydration. A lower or higher level of potassium can occur in combination with metabolic alkalosis or microcirculation disorders, and these can lead to symptoms such as depression, apathy, fatigue, drowsiness, confusion or even coma. Fineberg et al. [7] found that fluid/electrolyte disorders increase the risk of postoperative delirium in patients undergoing lumbar spine surgery. In the present study, we found that the risk of postoperative delirium in patients with electrolyte disturbances was 2.38 times that of individuals with normal electrolytes. Among them, sodium and calcium disorders, especially hyponatremia and hypocalcemia, played an important role in the occurrence of postoperative delirium. Similar to previous studies, we also found that a disturbance in sodium level was a very important risk factor for postoperative delirium. We also found the effect of hypocalcemia on postoperative delirium, which was less studied on postoperative delirium [31]. Therefore, the mechanism of hypocalcemia on postoperative delirium remains unclear. We believe that an effective balance of fluid and electrolytes is important for the prevention of postoperative delirium, which underscores the importance of perioperative management in elderly patients to reduce or avoid the occurrence of postoperative delirium.

## Limitations

This study has some limitations. First, although the phenomenon of postoperative delirium is universal, we have selected four common types of orthopedic surgery, and thus the research results probably cannot explain the incidence of delirium across all types of orthopedic surgery. Second, because this was a retrospective study, case selection could not comply with the principle of randomization. Third, we acknowledge that delirium is hard to be recognized. Although we have carefully checked medical records, we realize that mis-diagnosis might be present. Finally, many factors may be related to postoperative delirium; in this study, not all related factors were included, such as pain [32].

## Conclusion

In conclusion, the present study investigated the risk factors of postoperative delirium following four types of orthopedic surgery. Aging and postoperative electrolyte disorders (hyponatremia and hypocalcemia) were independent risk factors for postoperative delirium in patients undergoing these surgeries. Although the pathogenesis is not fully understood, postoperative delirium is considered a preventable disorder. Surgeons should actively identify risk factors and strengthen the perioperative management of patients to avoid the occurrence of postoperative delirium.

## Abbreviations

FHR: Femoral head replacement
IFIF: Internal fixation for intertrochanteric fracture
THA: Total hip arthroplasty
TKA: Total knee arthroplasty
